# New Developments in Cystic Fibrosis Airway Inflammation

**DOI:** 10.1155/2015/769425

**Published:** 2015-06-29

**Authors:** Nades Palaniyar, Marcus A. Mall, Christian Taube, Stefan Worgall, Hartmut Grasemann

**Affiliations:** ^1^Lung Innate Immunity Research Laboratory, Program in Physiology & Experimental Medicine, The Hospital for Sick Children Research Institute, Toronto, ON, Canada M5G 0A4; ^2^Department of Laboratory Medicine & Pathobiology, University of Toronto, Toronto, ON, Canada M5G 0A4; ^3^Institute of Medical Sciences, University of Toronto, Toronto, ON, Canada M5S 1A8; ^4^Department of Translational Pulmonology, Division of Pediatric Pulmonology and Allergy, and Cystic Fibrosis Center, Translational Lung Research Center Heidelberg (TLRC), University of Heidelberg, The German Center for Lung Research (DZL), 69120 Heidelberg, Germany; ^5^Department of Pulmonology, University Medical Center Leiden, Albinusdreef 2, 2333 ZA Leiden, Netherlands; ^6^Department of Pediatrics, Division of Pediatric Pulmonology, Allergy and Immunology, and Department of Genetic Medicine, Weill Cornell Medical College, New York, NY 10021, USA; ^7^Program in Physiology and Experimental Medicine, SickKids Research Institute and Division of Respiratory Medicine, Department of Paediatrics, The Hospital for Sick Children, University of Toronto, 555 University Avenue, Toronto, ON, Canada M5G 1X8

Cystic fibrosis (CF) is an autosomal recessive disease that is caused by mutations in the cystic fibrosis conductance regulator (CFTR) gene and usually presents with multiorgan involvement. Although life expectancy of people with CF has significantly improved since the discovery of the CFTR gene in 1989, chronic progressive lung function decline remains the major contributor to CF morbidity and mortality. CFTR is a cAMP-dependent Cl^−^ channel that in epithelial cells also functions as a regulator of other ion channels such as the amiloride-sensitive epithelial Na^+^ channel (ENaC). In CF airways, an imbalance in epithelial electrolyte secretion and absorption results in dehydration of the epithelial surface liquid layer, thickened secretions, defective mucociliary clearance, and a vicious cycle of mucous obstruction, infection, and inflammation. In recent years, there has been a tremendous advancement in the treatment options for patients with CF. With novel CFTR-targeting therapies, there are now opportunities to correct CFTR dysfunction and improve pulmonary function in some patients. However, airway inflammation remains a major factor in the disease and therefore this special issue is focused on the important role of airway inflammation in the development of chronic CF lung damage. The presented papers have been contributed by experts in the field and include both original research articles and state-of-the-art reviews.

One of the proposed pathophysiological mechanisms contributing to CF lung pathology is an imbalance between proteases and antiprotease in the airways. This concept has been recognized for decades but specific intervention studies so far have not been clinically successful. This important topic is thoroughly addressed in a review by M. S. Twigg et al. U. Müller et al., in their manuscript entitled “Changes of Proteases, Antiproteases, and Pathogens in Cystic Fibrosis Patients' Upper and Lower Airways after IV-Antibiotic Therapy,” present an interesting clinical research study on microbiological patterns and disparity of changes in protease/antiprotease imbalance between upper and lower airways of CF patients treated with antimicrobial agents.

In recent years, it has become apparent not only that ribonucleic acid (RNA) functions as a messenger in transcription but also that small noncoding RNA molecules (microRNA) play important roles in posttranscriptional regulation and modification. These microRNAs are now being studied in different diseases such as cancer, neurodegenerative conditions, and chronic lung diseases. The increasing body of literature on microRNAs in CF lung disease is summarized by P. J. McKiernan and C. M. Greene.

The regulatory mechanisms of CF airway inflammation are still not completely unraveled, but epithelial dysfunction seems to be pivotal for the development of CF-specific inflammatory responses. Current knowledge on CF epithelial function and host defense is summarized by F. Stanke. In addition, Z. Yu et al., in their original research study, provide novel data suggesting that ESE-1 may play a role in regulating CF airway inflammation via its effect on ICAM-1 expression.

Phosphoinositide 3-kinases are involved in cellular regulatory functions including cell growth, proliferation, differentiation, motility, survival, and intracellular trafficking. In this special issue, novel data are presented suggesting that phosphoinositide 3-kinase PI3K*γ* may play a role in the regulation of neutrophilic inflammation in CF. Indeed, M. Galluzzo et al. report results from experimental studies in the mouse that PI3K*γ* is implicated in chronic neutrophilic inflammation in CF-like lung disease. Their manuscript is entitled “Genetic Deletion and Pharmacological Inhibition of PI3K*γ* Reduces Neutrophilic Airway Inflammation and Lung Damage in Mice with Cystic Fibrosis-Like Lung Disease.”

Nitric oxide deficiency in CF airways is thought to be contributing to the increased risk of infections with certain pathogens including* Pseudomonas aeruginosa*. New experimental techniques for the assessment of the pulmonary L-arginine/nitric oxide metabolism in the mouse in vivo are described in the paper by H. Grasemann et al.: “Multitracer Stable Isotope Quantification of Arginase and Nitric Oxide Synthase Activity in a Mouse Model of Pseudomonas Lung Infection.”

We hope that this special issue not only will be useful to the interested reader by providing insight into new and important aspects related to CF airway inflammation but also may stimulate new interest and the development of novel research ideas and therapeutic avenues in this specific area.

*Nades Palaniyar* *Nades Palaniyar* *Marcus A. Mall* *Marcus A. Mall* *Christian Taube* *Christian Taube* *Stefan Worgall* *Stefan Worgall* *Hartmut Grasemann* *Hartmut Grasemann*

